# Association between dementia and mortality in the elderly patients undergoing hip fracture surgery: a meta-analysis

**DOI:** 10.1186/s13018-018-0988-6

**Published:** 2018-11-23

**Authors:** Jianzhong Bai, Pei Zhang, Xinyu Liang, Zhipeng Wu, Jingcheng Wang, Yuan Liang

**Affiliations:** 1 Dalian Medical University, Dalian, 116044 Liaoning China; 2 The second XiangYa hospital, Central South University, Changsha, 410011 China; 3 Clinical Medical College of Yangzhou University, Yangzhou, 225001 China

**Keywords:** Hip fracture, Mortality, Dementia, Meta-analysis

## Abstract

**Objective:**

This study was designed to verify the association between dementia and mortality in the elderly undergoing hip fracture surgery, and assessed the mortality of patients with dementia after hip fracture surgery.

**Material and methods:**

PubMed, Embase, and Web of Science were searched until April, 2018 without language restrictions. Two reviewers selected related studies, assessed study quality, and extracted data independently. Risk ratios (RRs) with 95% confidence intervals (CI) were derived using random-effects model throughout all analyses. The endpoints included 30-day, 6-month, 1-year, and more than 1-year mortality. This meta-analysis was performed following PRISMA statement and carried out by using stata14.0 software.

**Results:**

Dementia significantly increased postoperative mortality of patients suffered from hip fracture in 30-day [RR = 1.57, 95% CI (1.29, 1.90), P<0.00], 6-month [RR = 1.97, 95% CI (1.47, 2.63), P<0.00], 1-year [RR = 1.77, 95% CI (1.54, 2.04), P<0.00], and more than 1-year follow up [RR = 1.60, 95% CI (1.30, 1.96), P<0.00] respectively. The mortality of dementia patients after hip fracture surgery in 30-day [ES = 12%, 95% CI (8%, 15%)], 6-month [ES = 32%, 95% CI (17%, 48%)], 1-year [ES = 39%, 95% CI (35%, 43%)], and more than 1-year follow up [ES = 45%, 95% CI (32%, 58%)].

**Conclusions:**

Our meta-analysis demonstrated that the mortality of patients with dementia suffered from hip fracture surgery is 12%, 32%, 39%, and 45%, and dementia increased 1.57, 1.97, 1.77, and 1.60-fold mortality in patients undergoing hip fracture surgery in 30-day, 6-month, 1-year, and more than 1-year follow up respectively.

## Introduction

Hip fractures are being paid more attention due to higher morbidity and mortality. These patients usually have poor body compensatory capacity and comorbidities, such as dementia. Dementia is a syndrome characterized by persistent impairment in cognitive function or behavioral abnormalities. Due to the progress of aging society, dementia prevalence increases exponentially with age, it is estimated that the number of patients with dementia may reach 65.7 million in 2030 and 115.4 million in 2050 [1].

Dementia is not as fatal as deep vein thrombosis or pulmonary embolism in the short term, so it did not cause clinicians to pay enough attention, and it is unclear that the mortality of patients with dementia suffered from hip fracture surgery. Therefore, we performed a meta-analysis based on plenty of previous achievements in current research, to identify the effects of dementia on postoperative mortality of hip fracture, and the incidence of death in patients with dementia undergoing hip fracture surgery in 30 days, 6 months, 1 year, and more than 1 year follow up, and provided more convincing evidences for clinicians.

## Materials and methods

We carried out this meta-analysis according to the Preferred Reporting Items for Systematic Reviews and Meta-Analysis (PRISMA) statement [2] and Cochrane Collaboration guidelines strictly [3].

### Search strategy

We searched PubMed, Embase, and Web of Science until April, 2018, without language restrictions. In addition, the references of the included studies were manual searched to identify any additional articles. The following keywords were adopted in the database search: “hip fracture,” “mortality,” and “dementia.” The Boolean operators were used to combine them.

### Study selection and eligibility criteria

The inclusion criteria are as follows: (1) studies examined the relationship between dementia and mortality among elderly patients undergoing hip fracture surgery; (2) the sample size was more than 200; (3) study designs were cohort study; (4) the mean age is greater than 60 years. The exclusion criteria are as follows: (1) other types of fractures included; (2) studies provided insufficient data; (3) case report, review, commentary, and study just included an abstract; (4) dementia with other psychiatric disorders.

### Data extraction

Two reviewers (JZ B and P Z) independently extracted the following information from each study: first author, year of publication, country, study design, sample size, mean age, diagnostic criteria for dementia, and duration of follow-up (in-hospital, perioperative setting, 30-day, 6-month, 1-year and more than 1-year). Any discrepancies were resolved following discussion. All extracted data were entered into a predefined standardized Excel (Microsoft Corporation, USA) file carefully.

### Quality assessment

We evaluated the quality of studies by Newcastle-Ottawa Scale (NOS), which is a risk of bias assessment tool for observational studies [4]. This scale contains a number of answers per question ranging from two to five. High-quality responses earn a star, totaling up to nine stars. We set scores of 0–3, 4–6, and 7–9 for low, moderate, and high quality of studies, respectively [5].

### Statistical analysis

Statistical analyses were performed by using STATA 14.0 (StataCorp LP). The association between dementia and mortality was described as risk ratios (RR) with 95% confidence intervals (CI) by using random models. *P* ≤ 0.05 was regarded as statistically significant. Statistical heterogeneity was assessed by the Cochran Q test and quantified by the *I*^2^ statistic [6], and an *I*^2^ value greater than 50% indicates significant heterogeneity. When significant heterogeneity existed, heterogeneity analysis would be conducted to make a further explanation. We divided the follow-up time into four timepoints: 30-day, 6-month, 1-year, and more than 1-year follow-up. We classified the in-hospital and perioperative mortality as 30-day mortality.

## Results

### Study characteristics and quality assessment

**Fig. 1:**
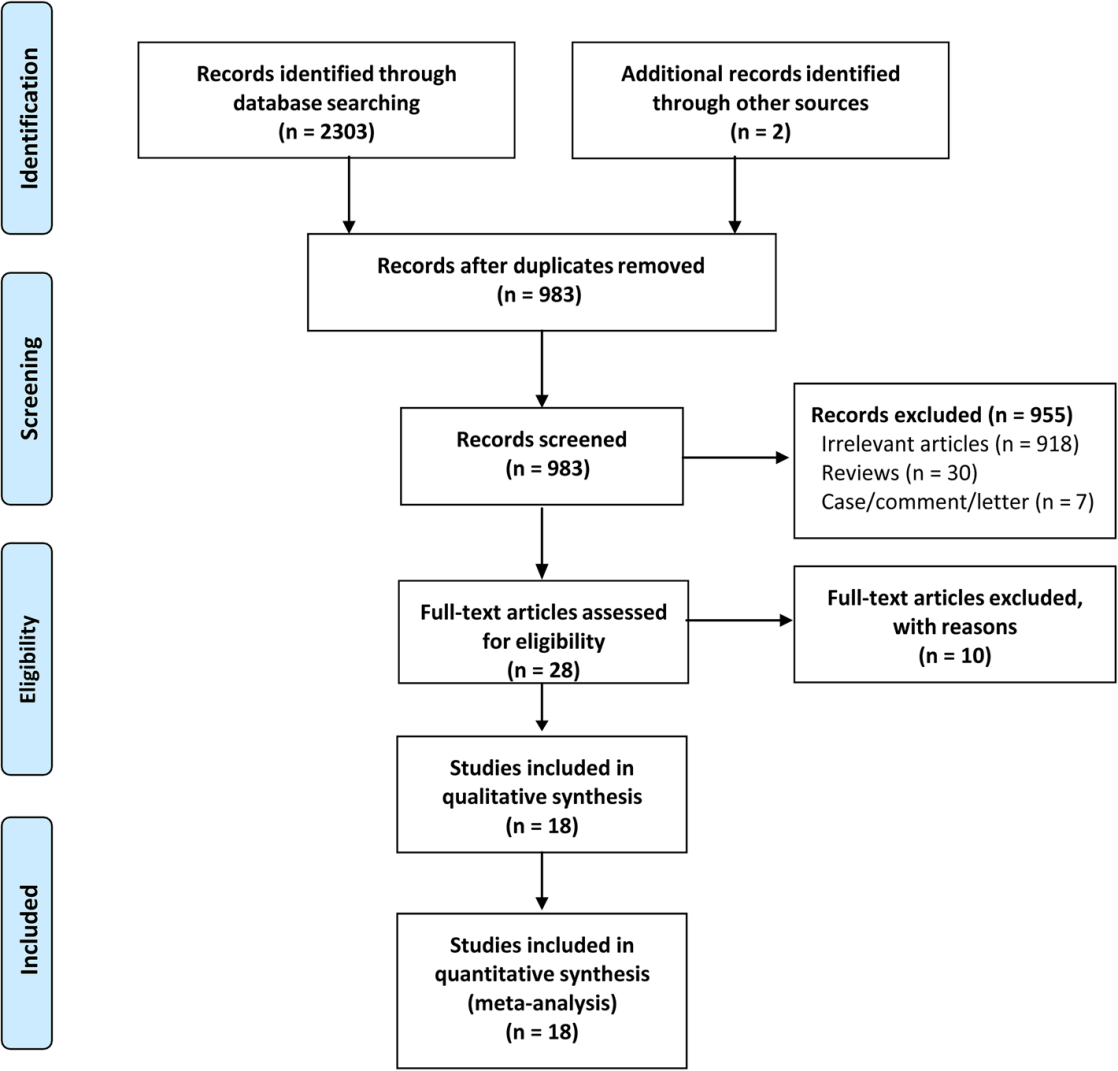
The flow chart of studies selecting

Two reviewers (JZ B and P Z) independently undertook the searches. Endnote X8 (version 18.0.0.10063) was used to remove duplicate studies. Additionally, we deleted irrelevant articles through the full text. Finally, 18 articles were included. The study selection process was shown in Fig. 1, and the main characteristics of the included trials were summarized in Table 1. Fourteen of 18 were prospective cohort studies [7–20], and four were retrospective cohort studies [21–24]. These studies enrolling 295,285 subjects were published between 2000 and 2018, with a sample size ranging from 272 to 134,144. The average scores of the quality assessment were 7.3 (range, 7–9). Details of the quality assessment were available in Table 2.

**Table 1:** Characteristics of the included studies

Studies	Country/design	Sample size	Mean age (years)	Follow-up (months)	30-day/6-month mortality	1-year/>1-year mortality
Holmes 2000 [8]	UK/PC	731	82.1	24	6 m D: 118/294 ND:93/409	1y D: 143/294 ND:121/409 > 1y D: 181/294 ND:159/409
Hannan 2001 [11]	USA/PC	571	≥70*	6	6 m D: 34/141 ND:43/430	NR
Mortimore 2008 [9]	USA/PC	674	81.1	24	NR	> 1y D: 49/120 ND:120/554
Pretto 2010 [10]	Switzerland/PC	272	84	12	NR	1y D:33/91 ND:24/172
Hershkovitz 2010 [12]	Israel /PC	376	82.3	24	NR	> 1y D: 59/237 ND:9/137
Julieb 2010 [14]	Norway/PC	331	85.1	12	NR	1y D: 14/50 ND:14/138
Frost 2011 [7]	Australia/ PC	1504	80.2	1	30d D: 20/214 ND:63/1290	NR
Khan 2013 [15]	UK/PC	467	80.2	1	30d D: 14/110 ND:21/357	NR
Kim 2013 [24]	Korea/RC	506	77.2	1	30d D: 0/40 ND:11/466	NR
Lee 2013 [16]	Korea/PC	790	77.8	72	NR	> 1y D: 51/78 ND:321/712
Cenzer 2016 [23]	USA/RC	857	84	12	NR	1y D: 49/133 ND:186/724
Tolppanen 2016 [20]	Finland/PC	134,144	79.9	36	NR	> 1y D: 22240/67072 ND:17771/67072
Ruggiero 2017 [17]	Italy/PC	514	83.1	12	NR	1y D: 20/102 ND:56/412
Reig 2017 [18]	Spain/PC	331	83	1	30d D: 9/92 ND:29/239	NR
Sheikh 2017 [19]	UK/PC	1356	81.4	1	30d D: 9/97 ND:109/1259	NR
Karres 2018 [21]	Netherlands/RC	1050	80	1	30d D: 15/131 ND:49/615	NR
Mitchell 2017 [22]	Australia /RC	27,888	≥ 65	12	NR	1y D: 2970/7132 ND:3977/19219
Jantzen 2018 [13]	Denmark /PC	122,923	80.6	12	30d D: 849/5230 ND:11372/117693	1y D:2334/5230 ND:32048/117693

*PC* prospective cohort study, *RC* retrospective cohort study, *D* dementia, *ND* non-dementia, *NR* no report; *calculated result

**Table 2:** Methodological quality assessment of included studies by Newcastle–Ottawa scales

Study	Selection				Comparability	Outcome			
	Exposed Cohort	Nonexposed Cohort	Ascertainment of exposure	Outcome of interest		Assessment of outcome	Length of follow-up	Adequacy of follow-up	Total score
Holmes et al.	*	*	*	*	*	*	*	*	8
Hannan et al.	*	*	*	*	*	*	–	*	7
Mortimore et al.	*	*	*	*	*	*	*	*	8
Pretto et al.	*	*	*	*	*	*	–	*	7
Julieb et al.	*	*	*	*	*	*	–	*	7
Hershkovitz et al	*	*	*	*	*	*	*	*	8
Frost et al.	*	*	*	*	*	*	–	*	7
Lee et al.	*	*	*	*	*	*	*	*	8
Khan et al.	*	*	*	*	*	*	–	*	7
Kim et al.	*	*	*	*	*	*	–	*	7
Cenzer et al.	*	*	*	*	*	*	–	*	7
Tolppanen et al.	*	*	*	*	**	*	*	*	9
Ruggiero et al.	*	*	*	*	*	*	–	*	7
Reig et al.	*	*	*	*	*	*	–	*	7
Sheikh et al.	*	*	*	*	*	*	–	*	7
Karres et al.	*	*	*	*	*	*	–	*	7
Mitchell et al.	*	*	*	*	*	*	–	*	7
Jantzen et al.	*	*	*	*	*	*	–	*	7

Single asterisk indicates 1 score; double asterisk indicates 2 scores, and dash indicates 0 scores

### Thirty-day mortality

**Fig. 2:**
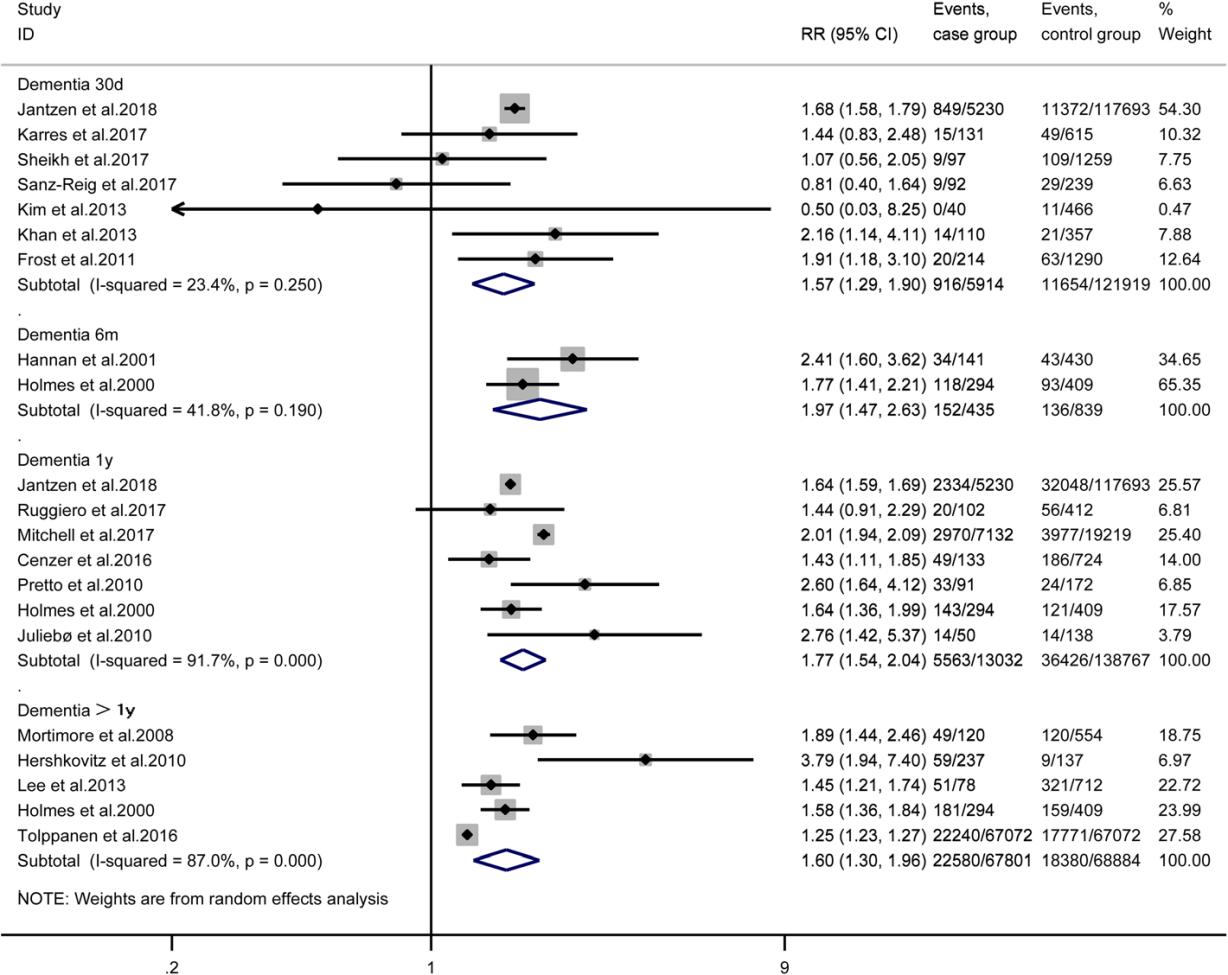
The effect of dementia on the mortality of patients after hip fracture surgery

**Fig. 3:**
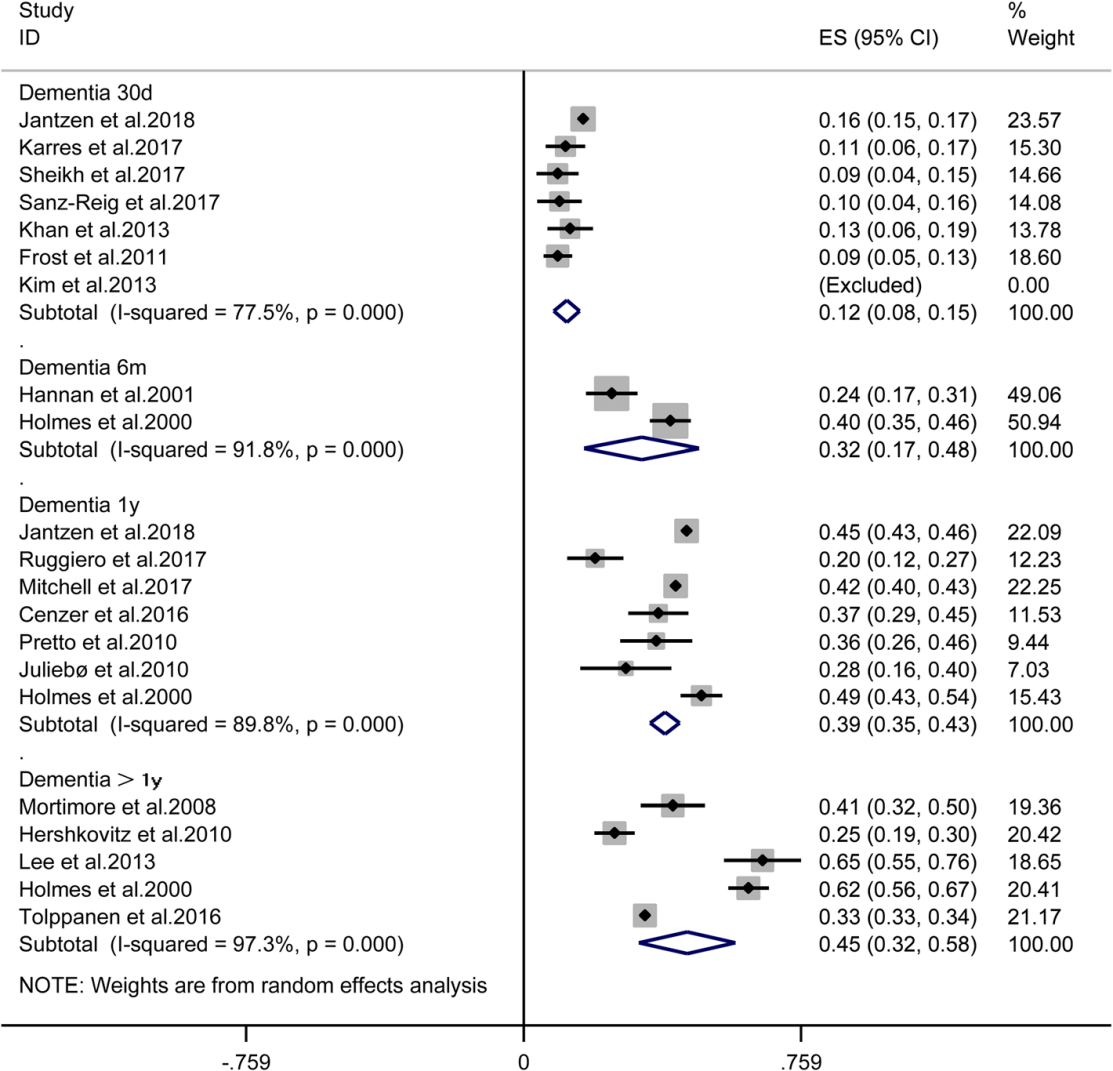
The mortality of dementia patients after hip fracture surgery

Seven studies [7, 13, 15, 18, 19, 21, 24] provided available data, and the pooled results suggested that dementia significantly increased the mortality of patients undergoing hip fracture surgery [RR = 1.57, 95% CI (1.29, 1.90), *P* < 0.001, *I*^2^ = 23.4%; Fig. 2], and the mortality of patients with dementia suffered from hip fracture surgery [ES = 12%, 95% CI (8%, 15%), *I*^2^ = 77.5%; Fig. 3].

### Six-month mortality

Only two studies [8, 11] provided available data in 6-month follow-up. The pooled outcomes suggested that dementia significantly increases the mortality of patients after hip fracture surgery [RR = 1.97, 95% CI (1.47, 2.63), *P* > 0.001, *I*^2^ = 41.8%; Fig. 2], and the mortality of the dementia patients suffered from hip fracture surgery [ES = 32%, 95% CI (17%, 48%), *I*^2^ = 91.8%; Fig. 3].

### One-year mortality

Seven studies [8, 10, 13, 14, 17, 22, 23] provided available data, and the pooled results suggested that dementia significantly increases the mortality of patients undergoing hip fracture surgery in 1-year follow up[RR = 1.77, 95% CI (1.54, 2.04), *P* < 0.001, *I*^2^ = 91.7%; Fig. 2], and the mortality of the dementia patients suffered from hip fracture surgery [ES = 39%, 95% CI (35%, 43%), *I*^2^ = 89.8%; Fig. 3].

### More than one-year mortality

Five studies [8, 9, 12, 16, 20] reported more than 1-year mortality. The pooled outcomes suggested that dementia significantly increases the mortality of patients undergoing hip fracture surgery [RR = 1.60, 95% CI (1.30, 1.96), *P* < 0.001, *I*^2^ = 87%; Fig. 2], and the mortality of the dementia patients suffered from hip fracture surgery [ES = 45%, 95% CI (32%, 58%), *I*^2^ = 97.3%; Fig. 3].

## Discussion

### Main findings

Our meta-analysis demonstrated that the mortality of patients with dementia suffered from hip fracture surgery is 12%, 32%, 39%, and 45%, and dementia increased 1.57, 1.97, 1.77, and 1.60-fold mortality in patients undergoing hip fracture surgery in 30-day, 6-month, 1-year, and more than 1-year follow-up. A previous meta-analysis included 75 studies, and involving 64,316 patients [25], the pooled outcomes demonstrated that the overall inpatient or 1 month mortality was 13.3%, 3 to 6 months was 15.8%, 1 year 24.5%, and 2 years 34.5% in generally patients, which mortality was significantly lower than patients with dementia who underwent hip fracture surgery.

### Comparison with other studies

To the best of our knowledge, there are three meta-analyses that described risk factors for postoperative mortality after hip fracture surgery [25–27], which dementia is one of these risk factors. However, these meta-analyses included fewer literatures for the indicator of dementia, and inconsistent follow-up time made conclusions less convincing. Therefore, we performed an update meta-analysis to evaluate the effects of dementia on postoperative mortality of patients undergoing hip fracture surgery and the incidence of death in patients with dementia undergoing hip fracture surgery in 30 days, 6 months, 1 year, and more than 1 year follow-up.

### Implications for clinical practice

Dementia has not received enough attention compared with deep vein thrombosis and pulmonary embolism in patients with hip fracture. However, our pooled results demonstrated that more than one third of people with dementia will die after hip fracture surgery in a 1-year follow-up and about one in two in more than 1-year follow-up. Patients with dementia usually have less activity and poor self-care ability, which increased postoperative complications, such as surgical site infection, urinary tract infection, and respiratory complications [28]. Therefore, orthopedists should attach great importance to this age-related disease, instead of just focusing on hip fracture problems. Besides, they should fully assess whether the dementia patients are suitable for surgical treatment after suffering from a hip fracture. Can surgical treatment improve the quality of life and prolong life span of patients, especially for patients with severe dementia? For patients with dementia who underwent hip fracture surgery, we should establish a multidisciplinary model of diagnosis and treatment to improve the postoperative outcomes [29]. Besides, nursing cares are necessary to prevent the adverse outcomes of dementia postoperatively [30], such as aspiration, pressure sore, deep vein thrombosis, and pneumonia. Further, rehabilitate exercises are needed to restore function as much as possible after discharge.

### Call for future studies

Our results show that patients with dementia have very high mortality rates after undergoing hip fracture surgery. Mortality will be higher for patients with severe dementia. It is unclear whether surgical treatment could improve quality of life and prolong life-span of patients with severe dementia. Until recently, there is no relevant meta-analysis or clinical guidelines to elaborate on this issue. We recommend that future studies should focus on which one is better for the treatment of patients with severe dementia who suffered from hip fracture, surgery, or conservative treatment. Besides, clinical decision makers should formulate relevant clinical guidelines to guide clinicians in the treatment of patients with dementia suffered from hip fracture.

### Heterogeneity analysis

The pooled results indicated that significant heterogeneity was found in some indicators. For the prevalence of death of patients with dementia after hip fracture surgery, (1) we conducted a sensitivity analysis through the deletion of Jantzen et al’s study [13], and the heterogeneity was reduced from 77.5 to 0.0% in 30-day mortality; (2) we excluded each study of the outcomes one at a time in 1-year mortality, but the heterogeneity was still very high. Unequal levels of regional medical care and significant sample size difference in the included studies may be the major sources of heterogeneity. For the effect of dementia on postoperative mortality, there was significant heterogeneity in 1-year mortality. The heterogeneity was reduced from 91.7 to 33.8% after the removal of Mitchell et al’s study [22]. Only two studies were included in 6-month mortality, which may be the main source of heterogeneity. In terms of more than 1-year mortality, the inconsistency of follow-up time and obvious sample size differences, which may be major sources of higher heterogeneity.

### Strengths and limitations of this meta-analysis

The major strengths of this study were the following: (1) our study was restricted to the elderly dementia patients undergoing hip fracture surgery, which were higher homogeneous and selective; (2) we designed our meta-analysis into four timepoints: 30-day, 6-month, 1-year, and more than 1-year follow up, and follow-up time is consistent; (3) we calculated the mortality of patients with dementia suffered from hip fracture surgery.

Limitations of this meta-analysis must be considered. First, although the patients we included in the study were elderly patients, it is difficult to make the confounding variables among the studies exactly the same. Second, we did not classify dementia into mild, moderate, and severe dementia because no related data were available in the original studies. Third, there was a great difference in the sample size of the included studies, which may affect the accuracy of our results to some extent. Fourth, significant heterogeneity was found in some outcomes. Last, although we conducted a comprehensive search of literature, it was hard to rule out the existence of publication bias.

## Conclusions

Our meta-analysis demonstrated that the mortality of people with dementia suffered from hip fracture surgery is 12%, 32%, 39%, and 45%, and dementia increased 1.57-, 1.97-, 1.77-, and 1.60-fold mortality in patients undergoing hip fracture surgery in 30-day, 6-month, 1-year, and more than 1-year follow-up. Future large-volume, well-designed studies, and the outcomes of multivariable analysis with extensive follow-up are awaited to confirm and update the findings of our analysis.

## Abbreviations

CI: Confidence interval
D: Dementia
ND: Non-dementia
NOS: Newcastle-Ottawa Scale
NR: No report
PC: Prospective cohort study
RC: Retrospective cohort study
RR: Risk ratio
